# Mr. Guthrie on the Operations for the Formation of an Artificial Pupil, &c.

**Published:** 1820-06-01

**Authors:** 


1. *A Treatise on the Operations for the Formation of an Artificial Pupil; in which the Morbid States of the Eye requiring them, are considered; and the Mode of performing the Operation, adapted to each peculiar Case, fully explained; with an Account of the Opinions and Practice of the different Foreign and British Authors who have written on the Subject. With Two Copper-plates.* By G. J. GUTHRIE, Deputy Inspector of Hospitals, Surgeon to the Royal Westminster Infirmary for Diseases of the Eye, &c. &c. One Vol. 8vo. pp. 209. London, 1819.

2. *A Treatise on Artificial Pupil, in which is described a Series of improved Operations for its Formation; with an Account of the Morbid States of the Eye to which such Operations are applicable.* By SIR WILLIAM ADAMS, Ophthalmic Surgeon to the Institution for the Cure of Blind Pensioners. Illustrated with coloured Engravings. One Vol. 8vo. pp. 134. With an Appendix, &c. London, 1819.

Rite secundarent *visus*: VIRG. ENEID.

A PERUSAL of the works prefixed to this Article, has led us into a train of reflections on the probable consequences which may accrue to medical science, should the present propensity to segregation and division of the Art continue or increase. The result of our observations and meditations is a decided conviction, that medical science will ultimately suffer, in *respectability* at least, should the said propensity subsist. The advocates for this subdivision of labour, however, bring forward specious arguments in their own favour, and one in particular, which it is no easy task to parry— namely, the grand division of Physic and Surgery, which time has sanctioned, custom confirmed, and even law enforced. Were we inclined to enter into this discussion, we think that we could shew the compatibility of a union of physic and surgery, without obstruction to the progress of either; but, at all events, we protest against the conclusion that, because one great division is made without inconvenience, or even with advantage, it follows that *subdivisions* may go on, ad infinitum, with similar consequences.

With respect to the Organ of Sight, which is the subject before us, we are quite satisfied: 1st. That its diseases cannot be properly studied without a regular and equal study of the whole human system; and, 2dly, that the operations on the eye may be learnt and practised by the general surgeon, so as to ensure equal, if not greater benefit to the patient, and success to the practitioner, than where there is an exclusive dedication to ophthalmic surgery set up.

By this we do not mean to argue against the propriety of exclusive institutions for diseases of the eye; we only wish to discountenance exclusive practice in that branch. It is not only consonant with sound theory, but we every day verify by observation, that when the human mind is *inordinately* devoted to the study of any one branch of natural knowledge, it becomes proportionally incapacitated for a general and comprehensive view of the circle of sciences. In the mechanical arts, indeed, this confinemment of mental and corporeal exertion to a single object is beneficial. A man may bring the manufacture of watch-springs to the greatest degree of perfection, without having any horological knowledge whatever. But not so in medicine. The eye, for instance, is subject to arthritic, rheumatic, syphilitic and other inflammations of constitutional origin, which cannot be treated successfully without a knowledge of these diseases as they affect other structures of the body. The function of the visual nerves, in common with all other functions, is daily deranged through sympathy with the digestive organs, the genital organs, and the intellectual organs. How then can the exclusive oculist pretend to treat such affections by means of his confined local specifics, and without an intimate knowledge of the original disorders, of which the ocular deviations are mere symptomatic phenomena ?

But the worst of all is this, that these exclusive professions open a wide door, as we observed in a former number, for the introduction of that trickery or quackery, which is the disgrace of medical science, and the surest method of sinking its professors in the public estimation. There is, alas! but too much of this despicable manœuvring, and wretched policy already in operation throughout the profession; and Heaven forbid that it should increase! As long as men continue to sacrifice the general interests and respectability of the Faculty at the shrine of selfish lucre, so long is it the duty of every zealous, and independent member of the profession to raise his voice against the indignity offered to medical science.

Our animadversions are not aimed at any individuals or class of individuals. We have nothing to do with private character or public controversy, unless they force themselves on our path; but we shall not be silent when we see the temple of knowledge profaned by the unhallowed footsteps of daring and mercenary intruders.

But to come to the subject of this article; we are glad to see ophthalmic institutions formed, and ophthalmic lectures delivered, by men who have distinguished themselves in the *general exercise of their profession,* and by their regular acquirements, in medical, surgical, pathological, and anatomical knowledge. By these men, and these institutions, ophthalmic *information* will be diffused, like every other species of medical or surgical knowledge, through the profession at large, and ophthalmic *practice* will be rescued from the hands of a monopoly, which, like all other monopolies, is an injury to many for the gain of a few.

Although we are far from commending those Compendia, or Omniana of medicine, which offer a whole circle of the science in the short compass of a portable volume, yet we cannot but think that distinct treatises on every coat, humour, and aperture of the eye, will have the effect of obstructing, instead of facilitating, the study of ophthalmic medicine and surgery. It is not to be expected that the general surgeon, and still less the general practitioner, can have access to voluminous publications on a single organ of the body; and we believe that the whole of what is necessary to be known as practical and elementary matter, on the subject of the eye, might be easily compressed into a single volume.

The works before us defy all attempts at analysis. In them are nearly as many divisions and subdivisions, on the single subject of artificial pupil, as there are genera, species, and varieties in the most complicated system of nosology! Of the two works, that of Sir William Adams is certainly more simple in its arrangement, and more capable of analysis; but it has the effect of constantly prepossessing us with the idea that the author's object is rather to enhance his own skill and dexterity, than to diffuse knowledge through the profession.

Non mundo sed sibi.

The liberal and scientific mind cannot bear the perpetual recurrence of such phrases as "my peculiar operations," and other egoisms of similar tendency. Nothing, in our opinion, can be worse taste or worse policy, in the end, than this practice, in any man who wishes to keep within the pale of the profession, or avoid the charge of Charlatannerie.

Mr. Guthrie's work is free from this defect; but the German minuteness with which it is split out into so many ramifications, makes it a hard matter, during its perusal, to carry simple and clear ideas in the mind, while so many complex images are presented to the eye and to the imagination.

"During the last three years (says Mr. Guthrie) that the Royal Westminster Infirmary for the Cure of Diseases of the Eye, has been established in Mary-le-bone Street, Piccadilly, my colleague, Dr. Forbes, the Physician to the Institution, and I, have devoted a part of our respective Courses of Lectures, on the Principles and Practice of Physic and Surgery, to the Diseases of the Eye, in order to render this branch of the healing art more intelligible to students; and to remove some of the difficulties which have prevented its being studied by practitioners. The limits of a course of lectures have not permitted us to enter so fully into historical details and theoretical opinions as we could have wished, although practical facts and opinions have never been neglected. The students have felt this, as well as the want of a work, on the Diseases of the Eye, which, whilst it noticed the doctrines and practice of the several writers on the subject, should state the facts, and impartially discuss the opinions of each; so as to obviate the necessity for purchasing several books on the same subject, merely because each author has chosen to recommend only his own practice, or methods of operating.

"On the subject of artificial pupil, there is no book in the English language which exhibits the opinions of foreign authors in a connected manner; and when they are hinted at, in some books, it is frequently in an erroneous manner; or the value of them is so much underrated or exaggerated, that it becomes very desirable the English reader should be better acquainted with them. To gain any thing like a tolerable acquaintance with the subject at present, a student must possess at least three books; Scarpa on the Diseases of the Eye; Mr. Gibson, and Sir W. Adams, on the Operations for Artificial Pupil; which is a serious inconvenience.

"With the hope of lessening the evil, this book was written. I have avoided entering into any unnecessary controversy, and where I have thought it right to combat the opinions of either the dead or the living, I have endeavoured to do it with liberality. I have stated the facts on both sides as fairly as I was able, and then drawn my inferences from them. I have also endeavoured to give to every one his own, and if I have failed towards any one, I shall be most willing to rectify the error on the slightest suggestion, whenever an opportunity shall be given me.

The observations which have been made on the different states of disease, and on the methods of operating, have been almost entirely from cases immediately under my own care, but which I have not detailed at length, because it would have tended to enlarge the work, without adding to its utility." Preface, x.

Mr. Guthrie's work is rendered exceedingly valuable to the English reader, by containing the various modes of operating for artificial pupil, recommended by Cheselden, Morand, Janin, Sharpe, Gendron, Guerin, Wenzel, Richter, Pellier de Quingsy, Plenck, Assalini, Buzzi, Forlenza, Arneman, Travers, Jurinne, Gleize, Demours, Scarpa, Schmidt, Sabatier, Maunoir, Donegana, Gibson, Sir W. Adams, Bëer, Reisenger, Himley, Buckhorn, Walther, Languenbeck, Frattini, Graefe, Jungken, Wagner, Embden, Dzondi, Zengs, Schlagintweit, Faure, Montain, Muter, Ryan, Quadri.

From this list, it is evident that no analysis of the work can be undertaken in a Medical Journal, with any prospect of success. Neither did we think it just to put the books in question into the hands of *ophthalmic* critics, for a *judicial* sentence; well knowing the trite but true apothegm, that "two of a trade can seldom agree."

We shall therefore introduce a single specimen from each of the works in question, leaving it to time and the public to award the prize of merit to the competitors.

Professor Bëer, of Vienna, has gained such celebrity in ophthalmic surgery, that we shall make our first extract from Mr. Guthrie's work, in order to introduce the said Professor's sentiments on the modes of forming an artificial pupil.

"The methods of operating may be classed under three principal heads.—Corotomia, corectomia, and corodialysis.

"The first has been very properly laid aside for a considerable time, because it is only applicable in a very few cases, and may now be fully dispensed with, in consequence of the adoption of the other two methods. Yet it sometimes happens that the operator, instead of separating the iris from the ciliary ligament, tears it, and thus accidentally performs corotomia. Yet no expert oculist, no man well versed in the art of operating, will have the least hesitation in preferring corodyalisis to corotomia, when he is perfectly at liberty to make a choice, provided corectomia is not, in some measure, contra-indicated.

"Agreeably to rule, the artificial pupil should always be formed near the inner angle of the eye, in the neighbourhood of the natural pupil. Yet, frequently, the operator is forced to make it towards the inferior, templar, or upper region, when the cornea is not staphylomatous; and the surgeon may, in these cases, think himself very fortunate to find a convenient place any where, on which he can operate with the requisite certainty.

"Corectomia is preferable in all cases where the lens is healthy, with the following exceptions. When the transparent part of the cornea is so circumscribed that a sufficient opening cannot be made in it to enable the operator to seize the iris with the hook or forceps, and to cut out a sufficiently large piece towards the ciliary ligament. Corectomia is also to be resorted to, when we are certain that the lymph coagulated in the posterior chamber after extraction, does not extend beyond the small ring of the iris, and is not connected with any opacity of the remaining capsule of the lens. The former may be ascertained from the natural colour and form of the larger ring of the iris; the existence of the latter may be suspected from the very imperfect perception of light, with respect to its particular modifications.

"The excision of the iris requires an incision at least one line in length, but which must run along the sclerotica, as near as possible to the edge of the cornea, that the operation may not be useless from subsequent opacity. In the second part of the operation, the act of excision may be attended by three different circumstances. When the iris is in no way improperly adherent to the cornea, it will be immediately protruded through the incision, by the gush of aqueous humour from the posterior chamber of the eye; of which the operator must instantly avail himself, by laying hold of the prolapsed part with a small cataract hook, and cutting it off as close as possible with a pair of Daviel's scissors, when the remaining part of the iris will immediately shrink back behind the cornea, and a well-formed pupil will be evident: or, when the iris is adherent, except at that part where the pupil is to be formed, (which may be discovered by viewing the eye laterally) the operator, after having made the incision, must introduce the small hook sideways, so as not to hook either the iris or the cornea, and then by an oblique direction of it, endeavour to lay hold of the pupillary edge of the iris, and drawing it out, cut it off, as before directed; by which he not only increases the size of the natural pupil, so that it now extends behind the transparent part of the cornea, but greatly augments the power of vision, because the rays of light will fall more upon the centre, and less upon the edge of the crystalline lens. Thirdly, and finally: The iris may be connected by its pupillary edge to the cornea, even at the place where the pupil is to be formed. In this case, it must be laid hold of by the hook near its larger ring, or if that tears out, a pair of fine-pointed and indented forceps are to be introduced, and the iris thus torn, is to be drawn out, if possible, and the piece cut off; but if it cannot be drawn out, the piece seized by the forceps must be cut off within the edge of the incision; because a perseverance in the attempt to draw the iris more forcibly out, will tear it, in all probability, in a manner highly prejudicial to the success of the operation. The healthy lens and capsule can never be injured, provided the patient is steady, and the operator sufficiently dexterous. The latter method, viz. with the forceps, must at once be resorted to, when we wish to form an artificial pupil, after a previous extraction of the cataract; but this is only practicable when the capsule is not adherent, and when there is but a small quantity of coagulable lymph in the posterior chamber, and not then extending beyond the smaller circle of the iris, towards the ciliary ligament.

"The separation of the iris from the ciliary ligament is only indicated—first, when after an extraction or reclination of the cataract, the lymph thrown out in the posterior chamber, in consequence of inflammation, extends towards the ciliary ligament, far beyond the smaller ring of the iris, which may be ascertained with tolerable accuracy, from the alienation of the colour of the larger ring of the iris, and a somewhat imperfect perception of light. Secondly, when we have to deal with a secondary capsular, or capsulo lenticular cataract, which is adherent to the iris; or, with an opacity of the pupil, resulting from the deposition of matter or blood; and with which there is however, as is sometimes the case, a distinct perception of light, and no symptoms decidedly unfavourable to the operation. Thirdly, and finally; when the cornea is so marked by the cicatrixes of ulcers, or is incurably opaque, and to such an extent, that it cannot properly be opened with the knife, so as to enable us to undertake the operation of excision.

"In the two first cases, in order to perform corodialysis quickly and successfully, Schmidt's lanced-shaped curved needle (supposing that the pupil is to be performed towards the inner angle of the eye) is to be introduced into the anterior chamber, a good half line from the outer edge of the cornea, the convexity of the needle being turned towards the iris. It is then to be carried without touching either the cornea or iris, to the inner edge of the cornea, when the point of it is to be pushed so deeply into the iris, within the distance of the eighth part of a line from its outer margin, that it may be firmly hooked. A double motion is then to be executed with the handle of the needle; for, the handle is to be raised, so as to press the point of the needle into the iris and vitreous humour, whilst the needle is, at the same moment, to be withdrawn, but not entirely out of the eye. The point of it is now to be loosened from the iris, and the eye examined, to see whether the separated iris does not again return towards the ciliary ligament, which is unfortunately but too generally the case. If the iris shews the least disposition to return, or if the pupil thus made be too small, the iris is to be again laid hold of with the point of the needle, at the upper or lower angle of the new pupil, and the operation of separation is to be repeated; when the artificial pupil will certainly appear, and remain as large as can be wished. But, if the coagulated albumen and lymph in the posterior chamber of the eye, really extend to the ciliary ligament, the iris may be stretched and pulled about in an incredible manner, but can never be separated; and the attempt at making an artificial pupil will not succeed, unless by a fortunate accident, the iris and the pseudo membrane, which is behind it, should be torn asunder, and give rise in this manner to a pupil of sufficient dimensions. In the third case, Schmidt's needle must be introduced into the eye through the sclerotica, as in the reclination of the cataract, and carried on with its concave surface turned to the iris, towards that part of the ciliary ligament where the pupil is to be formed. It is then, as recommended by Schmidt, to be pushed from behind, forwards into the iris, about the eighth part of a line from the ciliary ligament, in order to lay hold of, and to separate it sufficiently by one or two attempts, which is exactly the reverse of the method recommended in the former cases. In either instance, the lens, whether transparent or adherent, will naturally be displaced at the moment of separation by the double movement of the needle, and consequently be so far out of the limits of the artificial pupil, that it can never be injurious to vision, even on its becoming opaque at a subsequent period, which will, and must inevitably be the case.

"It is now no longer to be doubted, from recent experiments, made upon persons totally blind, that corodialysis, performed with Reisinger's hooked forceps, has, in many cases, great advantages over this method; but, whether it is such as to deserve a place in this work, as a prototype of operative proceeding, must be decided by further experience." 38—45.

Mr. Guthrie, as a teacher and an author, maintains his own dignity, and that of the profession to which he belongs. The motto of his work might fairly be ——

Non sibi sed mundo.

And we venture to predict, that this is the best policy in the long run. However prone human nature may be to selfishness and finesse, nothing can be more certain, than that every man despises these propensities in another. We throw out these general reflections as Fortune distributes her favours —blindfold. No man need, or will apply them to himself, or feel offended thereby, unless they should happen to touch a nerve of *morbid* sensibility in the system of his personal economy.

The following operation is that which Sir William Adams recommends for simple contraction or obliteration of the pupil, succeeding extraction, depression, absorption of the crystalline lens, or accidents occurring to the eye from sharp instruments puncturing the capsule, &c.

"The patient being seated as in the operation for cataract, and the eye being steadied, either by the finger of the assistant who supports the upper lid, or by gentle pressure made with my concave speculum, the iris scalpel already described, with its edge turned backwards, must be introduced through the coats of the eye at their external part, about a line behind the iris, and in the transverse diameter of the latter membrane. The point of the instrument should then be made to penetrate through the iris, into the anterior chamber, in a line with its central diameter, and somewhat less than one-third of the width of that membrane, from its ciliary margin. The iris scalpel is then to be carried cautiously through the anterior chamber towards the inner canthus, keeping its edge in contact with the iris (in order to prevent the point from piercing the internal part of the cornea,) until it has traversed more than two-thirds the width of the iris, when it should, with great care, be drawn backwards, almost out of the eye, making the most delicate pressure with the edge of the instrument against the iris, lest it should be detached from the ciliary ligament. If the division of the iris is not effected to a sufficient extent during the first effort, the iris scalpel should be again carried forward, and withdrawn in a similar manner. This is to be repeated as often as may be necessary, to effect a division of the iris, to the extent of a third part of its diameter." 35.

Into the subject of the Postscript and Appendix to Sir William Adams's work, we shall not enter. The Medical Journals, on both sides of the Tweed, have suffered in character by permitting themselves to become the vehicles of disgraceful controversy; and even were our own Journal open to papers of this kind, (which, thank God, it is not) we should hardly be blind enough to run upon such dangerous shoals. And as we open no Arena for medical combatants to abuse each other, and injure the profession in the eyes of the world, so will we never lend ourselves to a party, or sully our pages with the virulent invectives of contending interests.

Ab honesto nulla re deterribitur, ad turpia, nulla spe invitabitur.

SENECA.

*Pharmacologia;*

